# Attention mechanism based multi-sequence MRI fusion improves prediction of response to neoadjuvant chemoradiotherapy in locally advanced rectal cancer

**DOI:** 10.1186/s13014-023-02352-y

**Published:** 2023-10-27

**Authors:** Xuezhi Zhou, Yi Yu, Yanru Feng, Guojun Ding, Peng Liu, Luying Liu, Wenjie Ren, Yuan Zhu, Wuteng Cao

**Affiliations:** 1 College of Medical Engineering, Xinxiang Medical University, Xinxiang, 453003 Henan China; 2 Engineering Technology Research Center of Neurosense and Control of Henan Province, Xinxiang, China; 3 Henan International Joint Laboratory of Neural Information Analysis and Drug Intelligent Design, Xinxiang, China; 4 Department of Radiation Oncology, Zhejiang Cancer Hospital), Institute of Basic Medicine and Cancer (IBMC), The Cancer Hospital of the University of Chinese Academy of Sciences, Chinese Academy of Sciences, Hangzhou, Zhejiang China; 5 Zhejiang Key Laboratory of Radiation Oncology, Hangzhou, China; 6 Department of Radiology, Institute of Basic Medicine and Cancer (IBMC), The Cancer Hospital of the University of Chinese Academy of Sciences (Zhejiang Cancer Hospital), Chinese Academy of Sciences, Hangzhou, Zhejiang China; 7 Department of Radiology, The Sixth Affiliated Hospital, Sun Yat-sen University, Guangzhou, 510655 Guangdong China; 8 Guangdong Provincial Key Laboratory of Colorectal and Pelvic Floor Diseases, The Sixth Affiliated Hospital, Sun Yat-sen University, Guangzhou, Guangdong China

**Keywords:** Attention, Fusion, Neoadjuvant chemoradiotherapy, Response prediction, Locally advanced rectal cancer

## Abstract

**Background:**

Accurate prediction of response to neoadjuvant chemoradiotherapy (nCRT) is very important for treatment plan decision in locally advanced rectal cancer (LARC). The aim of this study was to investigate whether self-attention mechanism based multi-sequence fusion strategy applied to multiparametric magnetic resonance imaging (MRI) based deep learning or hand-crafted radiomics model construction can improve prediction of response to nCRT in LARC.

**Methods:**

This retrospective analysis enrolled 422 consecutive patients with LARC who received nCRT before surgery at two hospitals. All patients underwent multiparametric MRI scans with three imaging sequences. Tumor regression grade (TRG) was used to assess the response of nCRT based on the resected specimen. Patients were separated into 2 groups: poor responders (TRG 2, 3) versus good responders (TRG 0, 1). A self-attention mechanism, namely channel attention, was applied to fuse the three sequence information for deep learning and radiomics models construction. For comparison, other two models without channel attention were also constructed. All models were developed in the same hospital and validated in the other hospital.

**Results:**

The deep learning model with channel attention mechanism achieved area under the curves (AUCs) of 0.898 in the internal validation cohort and 0.873 in the external validation cohort, which was the best performed model in all cohorts. More importantly, both the deep learning and radiomics model that applied channel attention mechanism performed better than those without channel attention mechanism.

**Conclusions:**

The self-attention mechanism based multi-sequence fusion strategy can improve prediction of response to nCRT in LARC.

**Supplementary Information:**

The online version contains supplementary material available at 10.1186/s13014-023-02352-y.

## Background

Neoadjuvant chemoradiotherapy (nCRT) followed by total mesorectal excision (TME) is widely recommended as the standard treatment strategy for locally advanced rectal cancer (LARC) [1, 2]. Response to nCRT is a favorable indicator of good prognosis. Approximately 50-60% of patients with LARC are downstaged and about 20% show pathological complete response after nCRT [1, 3, 4]. However, compared with good responders to nCRT, poor responders tend to have higher local recurrence and recurrence-free survival rate [3]. These poor responders may benefit little from nCRT, but still experience toxic effects of treatment such as diarrhea, nausea, hematological infection, and fever [5, 6]. Therefore, pretreatment prediction of the response to nCRT is of great importance for devising appropriate personalized treatment plans.

Magnetic resonance imaging (MRI) is the most important imaging approach for assessment of treatment response in LARC patients [7]. Pretreatment MRI features, such as tumor volume, tumor height, depth of tumor penetration, and absence of extramural venous invasion, are reported to be associated with a better response to therapy [8, 9]. However, visual assessment of MRI features is limited by subjectivities and relies on experiences of radiologists [10]. Thus, pretreatment accurate identification of nCRT poor responders still remains challenge other than by pathologic evaluation after completing neoadjuvant therapy.

Recently, radiomics and deep learning has drawn great attractions due to the superiority of quantifying imaging phenotype that associated with the underlying tumor pathological character from MRI or other imaging modalities beyond visual interpretation [11]. A growing number of studies aimed at prediction of treatment response have supported more effective performance basing on multiparametric MRI [12–15]. To predict poor responders, Zhou et al. developed a radiomics model based on pretreatment apparent diffusion coefficient (ADC) map, T1 weighted- (T1w), T1 contrast-enhanced (T1c) and T2 weighted (T2w) MRI with an area under the receiver-operating characteristic (ROC) curve (AUC) value of 0.773 [16]. However, all above studies just combined multiparametric MRI in a very simple strategy, which concatenated radiomic features or deep learning features according to different imaging sequences for further feature selection or fully connected network.

The self-attention mechanism has been widely used in various medical imaging analyses such as segmentation [17], classification [18] and survival prediction [19]. Without any explicit supervision, self-attention mechanism can learn to focus on important features via training end-to-end together with the original convolutional neural networks (CNN) backbone. Squeeze-and-Excitation (SE) [20] network is a prominent approach that focuses on channel attention, which natural fit for multi-parametric fusion. Therefore, the present multicenter study aims at investigating whether SE-based channel attention mechanism is better than feature concatenation on the task of treatment response prediction in LARC.

## Methods

### Patients

**Fig. 1:**
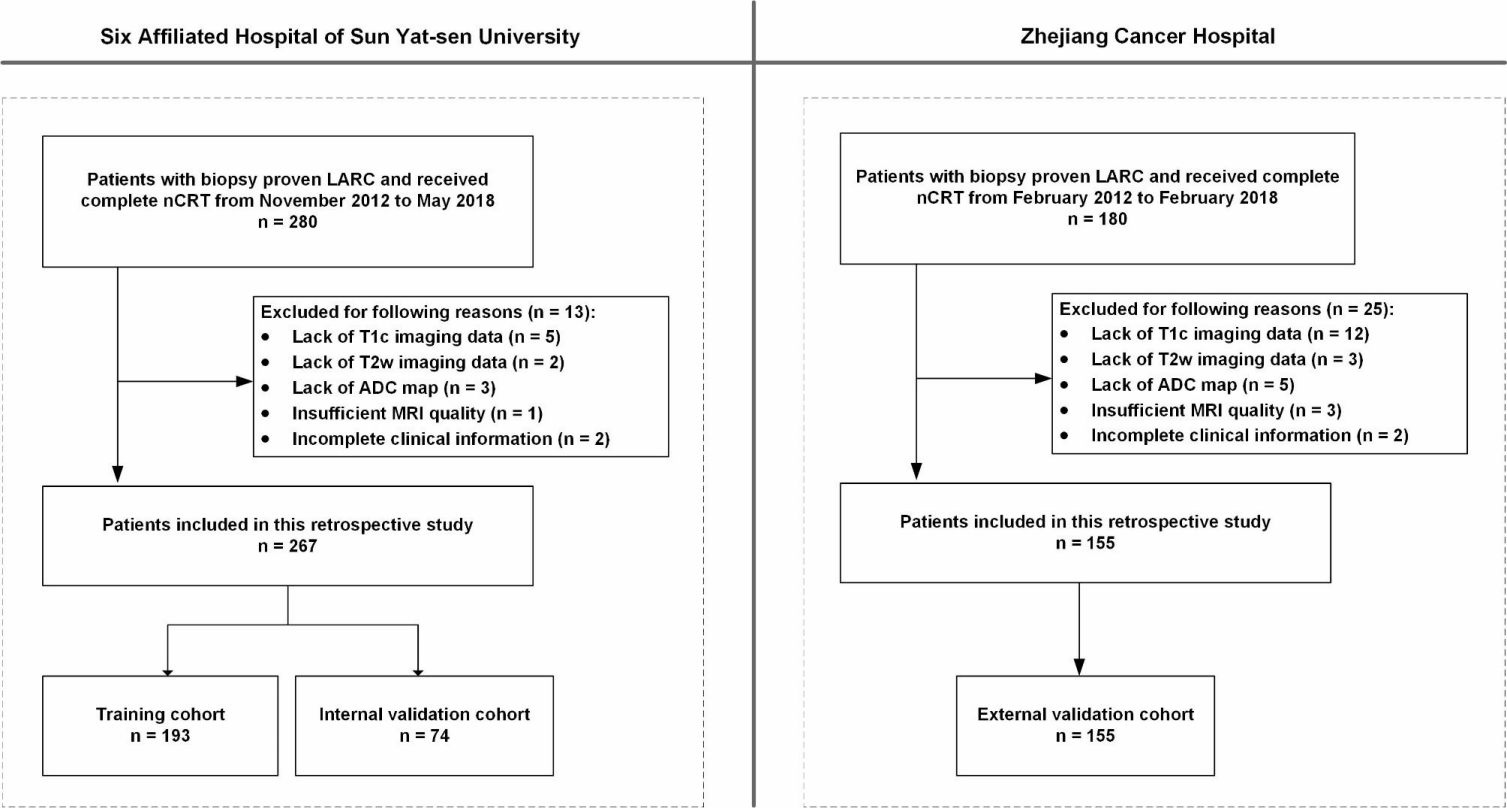
Recruitment process of the present study. nCRT, neoadjuvant chemoradiotherapy; LARC, locally advanced rectal cancer; ADC, apparent diffusion coefficient; T1c, T1 contrast-enhanced; T2w, T2 weighted

This retrospective multicenter study was approved by the Ethics Committee of the Six Affiliated Hospital of Sun Yat-sen University (SAHSYU) and that of the Zhejiang Cancer Hospital (ZCH). The requirement for informed patient consent was waived. This study included eligible patients who were diagnosed with LARC by multiparametric MRI examination and received standard chemoradiotherapy treatment between February 2012 and May 2018, as shown in Fig. 1. Patients who met the following exclusion criteria were removed from the analysis: (i) lack of pathologic treatment response evaluation after treatment; (ii) lack of MRI sequence including T2w, T1c and ADC; (iii) insufficient MRI quality due to bowel peristalsis-related artifacts; (iv) lack of clinical information including sex, age, clinical T stage (cT-stage), clinical N stage (cN-stage) and carcinoembryonic antigen (CEA; cutoff ≥ 5 ng/ml, < 5 ng/ml) blood level.

### Neoadjuvant chemoradiotherapy

All enrolled patients underwent preoperative treatment with five cycles of infusional fluorouracil (leucovorin 400 mg/m2 intravenously followed by fluorouracil 400 mg/m2 intravenously and fluorouracil 2.4 g/m2 by 48-h continuous intravenous infusion) and concurrent radiation treatment. Radiotherapy was delivered at 1.8 to 2.0 Gy daily Monday through Friday for a total of 23 to 28 fractions over 5 to 6 weeks and a total dose of 46.0 to 50.4 Gy.

### Assessment of response to nCRT

The pathologic treatment response after nCRT was evaluated based on TME resection specimens, according to the four-tier American Joint Committee on Cancer (AJCC) Cancer Staging tumor regression grade (TRG) systems [21]. The four TRG groups were as follows: TRG 0, no residual tumor cells; TRG 1, single tumor cell or small group of tumor cells; TRG 2, residual cancer with desmoplastic response; TRG 3, minimal evidence of tumor response. According to the AJCC TRG systems, patients were separated into 2 groups: poor responders (TRG 2, 3) versus good responders (TRG 0, 1).

### MRI Acquisition and Tumor Segmentation

All patients from Six Affiliated Hospital of Sun Yat-sen University underwent T2w, T1c and ADC MRI scans before the nCRT with 1.5 Tesla MRI (Optima MR 360, GE Medical Systems, USA) using an eight-element body array coil with fixed image protocols. All patients from Zhejiang Cancer Hospital underwent T2w, T1c and ADC scans before the nCRT with 3.0 Tesla MRI (Verio 3.0T MR, Siemens Medical Systems, Germany). Parameters for MRI acquisition were shown in supplementary Table S1. Before further analysis, all axial slices were normalized into 1 mm × 1 mm pixel spacing. The region of interest (ROI) was manually delineated around the tumor outline via the itk-SNAP software (www.itksnap.org) on the axial slice with the largest lesion cross-section of T2w images by one gastrointestinal radiologist (reader 1) with 10 years of experience, and was then copied onto the corresponding slice of T1c and ADC images. In addition, the segmentation was examined by another gastrointestinal radiologist (reader 2) with 30 years of experience. If reader 2  found any discrepancies between the segmentation and the actual tumour outline, he provided feedback to reader 1 for consultation. The segmentation was then adjusted in consultation with reader 1. The main concern is to outline the tumour area avoiding gas in the intestinal lumen, fat or other structures around the lesion area, etc. This is done to ensure that the outlined area corresponds to the tumour.

### Experimental design

In the present study, two kinds of channel attention fusion approaches were conducted for comparison, which consist of a hand-crafted radiomic features-based fusion and an image-based fusion. Radiomic features were firstly extracted from the ROIs of T2w, T1c and ADC images using PyRadiomics version 2.2.0 [22]. A total of 372 radiomics features were extracted from each imaging sequence in this study, of which 93 were extracted from original images and 279 were extracted from three scale Laplacian of Gaussian (LoG) transformed images. Among the 93 features extracted from original images, 18 were first-order statistics and 75 were textural features originated from Gray Level Cooccurence Matrix (n = 24), Gray Level Run Length Matrix (n = 16), Gray Level Size Zone Matrix (n = 16), Gray Level Dependence Matrix (n = 14) and Neighbouring Gray Tone Differnece Matrix (n = 5). Then ROIs of the three imaging sequences were cropped by an 80 × 80 bounding rectangle as a three-channel input for image-based fusion.

**Fig. 2:**
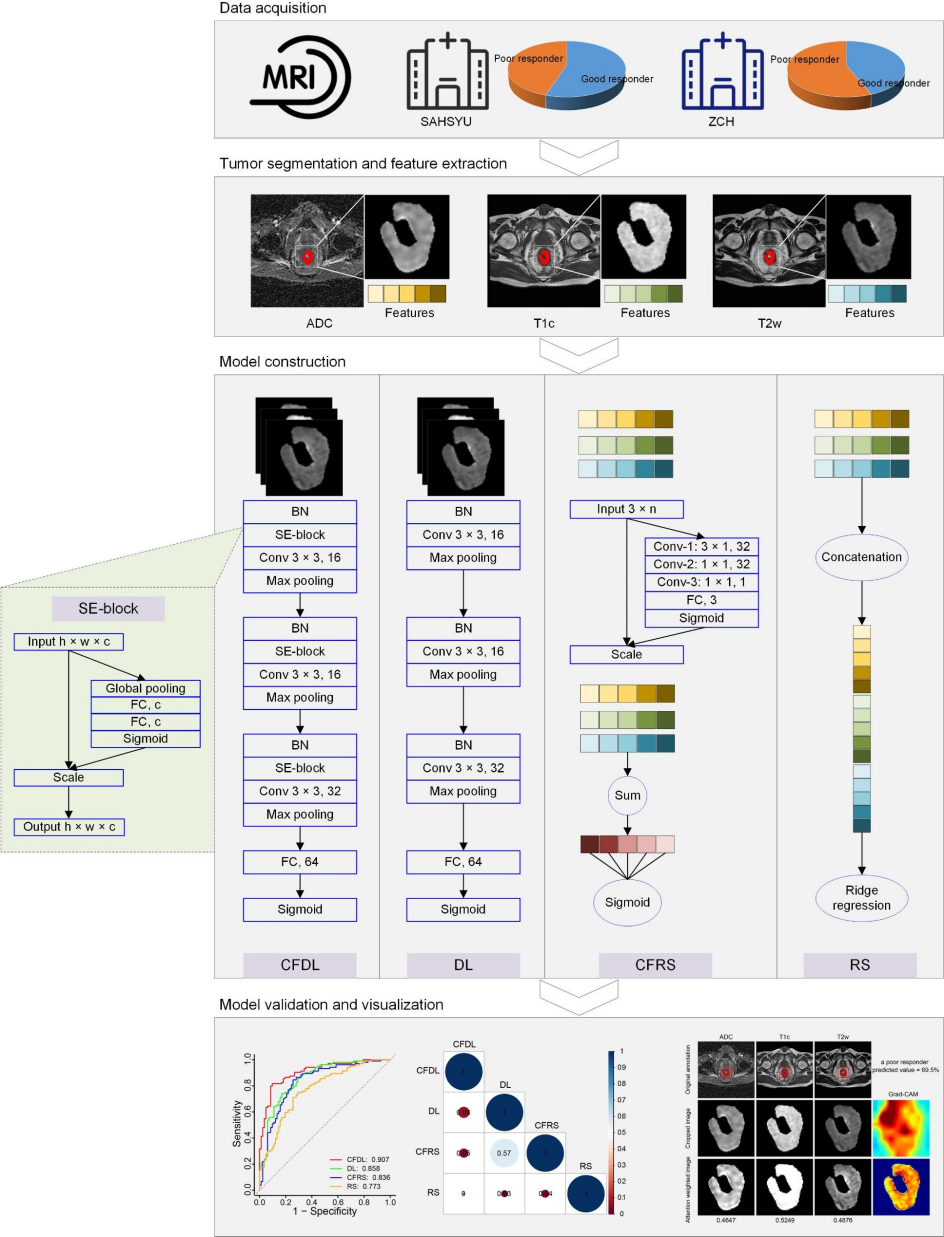
Experimental design of the present study. SAHSYU, Six Affiliated Hospital of Sun Yat-sen University; ZCH, Zhejiang Cancer Hospital; ADC, apparent diffusion coefficient; T1c, T1 contrast-enhanced; T2w, T2 weighted; BN, batch normalization; Conv, convolution; FC, fully- connected; SE, Squeeze-and-Excitation.

To realize radiomic features-based channel fusion (hereinafter referred to as CFRS.), a simple self-attention module was designed using 2D convolution kernel as shown in Fig. 2. For each patient, n features were extracted from each modality of MRI, generating a feature matrix of size 3 × n as the input for the self-attention module. The self-attention module consists of three convolutional layers. The first convolutional layer has 32 filters of size 3 × 1, followed with 32 filters of size 1 × 1 in the second convolutional layer. The third convolutional layer has one filter of size 1 × 1, followed with fully connected layer consisting of 3 elements activated with sigmoid function. Then the output of the fully connected layer was set as 3 self-attention weighting coefficients of the three channels, resulting a n-dimensional channel-fused radiomic feature. At last, the fused feature was sent to a fully connected layer consisting of 1 element activated with sigmoid function to predict the probability of poor response.

To realize image-based channel fusion (hereinafter referred to as CFDL), SE module [20] was used as shown in Fig. 2. Then a shallow CNN model consisting of SE module and 3 convolutional layers followed by 2 fully connected layers was constructed for poor responder prediction. The detailed parameters of this model were also shown in Fig. 2.

The comparative performance tests were conducted for the model with/without channel fusion. Concretely, a traditional radiomics model (hereinafter referred to as RS) was built by ridge regression using the three-sequence concatenated features, and a deep learning model without SE module (hereinafter referred to as DL) using three-sequence concatenated images was also built for comparison. There 70% of patients in SAHSYU were randomly selected for fitting the model parameters, and the rest 30% of patients in SAHSYU were used for internal validation. The loss function of ridge regression can be expressed as blow:

$$f\left(w\right)=\sum _{i=1}^{m} {\left({y}_{i}-{x}_{i}^{T}w\right)}^{2}+\lambda {\parallel w\parallel }_{2}^{2}$$

Where ${y}_{i}$ is the label of patient $i$, $m$ is the number of patients, ${x}_{i}^{T}$ is the feature vector of the patient$i$, $w$ is the coefficient of the feature, and λ is the regularization parameter. To determine the optimal value of the regularization parameter $\lambda$, we performed 5-fold cross-validation in the training cohort and tested values of $\lambda \in$(0.001, 1) with a step size of 0.001 at the same time. The $\lambda$ value that maximized the average AUC of the cross-validation was selected as the optimal regularization parameter.

Gradient (Grad)-CAM [23], a flexible method can interpret arbitrary layers of a CNN without the need of any architecture modifications, was used to identify classification-relevant CNN features in the present study. Heatmap generated by Grad-CAM was used for individually visualizing the critical discriminating image regions.

### Statistics

R software (version 3.6.2) was used for all statistical analyses. All tests were 2-sided, and P values < 0.05 indicated statistical significance. The difference of clinical variables between two groups was compared by using the Mann-Whitney rank-sum test or adopting the chi-square (χ2) test. The sensitivity, specificity, positive predictive value (PPV), negative predictive value (NPV), accuracy, and AUC of different models were calculated. Delong test was performed to compare the difference of predictive performance for two arbitrary models. Multivariate logistic regression analysis was performed to assess if the result predicted by each of the four constructed models was an independent predictive factor when considering the other three constructed models and clinical variables such as sex, age, clinical T stage, clinical N stage and CEA blood level.

## Results

### Patients

A total of 422 patients were finally included in this study. The clinical characteristics of these patients were shown in Table 1. The dataset with a sample size of 193 randomly separated from the SAHSYU was used as the training cohort, and dataset from the ZCH were used as the external validation cohort. The rate of poor responders was 55.1% (147/267) and 72.3% (112/155) in the SAHSYU and ZCH cohorts, respectively. The proportion of poor responders varies a lot (p < 0.001) in the two cohorts. CEA and clinical T stage were also significantly (p < 0.05) different between the two cohorts. Besides clinical T stage in ZCH, the other variables did not show significant difference between poor responders and good responders in each of the two cohorts.

**Table 1:** Clinical characteristics of patients in two hospitals

Characteristics	SAHSYU (n = 267)				ZCH (n = 155)			P
	**Poor response** **(n = 147)**	**Good response** **(n = 120)**	***p***		**Poor response** **(n = 112)**	**Good response** **(n = 43)**	***p***	
Age, years	54.8 ± 12.4	53.0 ± 11.4	0.180		56.1 ± 10.9	56.6 ± 8.0	0.803	0.124
Sex			0.131				0.968	0.673
Male	117 (79.6%)	86 (71.7%)			83 (74.1%)	32 (74.4%)		
Female	30 (20.4%)	34 (28.3%)			29 (25.9%)	11 (25.6%)		
CEA			0.07				0.583	0.01
Positive	65 (44.2%)	40 (33.3%)			57 (50.9%)	24 (55.8%)		
Negative	82 (55.8%)	80 (66.7%)			55 (49.1%)	19 (44.2%)		
cT			0.113				0.038	< 0.001
T2	7 (4.8%)	14 (11.7%)			1 (1%)	0		
T3	112 (76.2%)	84 (70.0%)			64 (57.1%)	34 (79.1%)		
T4	28 (19%)	22 (18.3%)			47 (41.9%)	9 (20.9%)		
cN			0.977				0.676	0.076
N0	35 (23.8%)	29 (24.2%)			17 (15.2%)	7 (16.3%)		
N1	55 (37.4%)	46 (38.3%)			50 (44.6%)	22 (51.2%)		
N2	57 (38.8%)	45 (37.5%)			45 (40.2%)	14 (32.5%)		

Continuous data are given as mean ± standard deviation

P values for categorical variables were from chi-square test analysis

P values for continuous variables were from Mann-Whitney rank-sum test analysis

SAHSYU, Six Affiliated Hospital of Sun Yat-sen University; ZCH, Zhejiang Cancer Hospital; cT, clinical T stage; cN, clinical N stage

### Model construction and validation

The RS model constructed by three-sequence feature concatenation and ridge regression achieved an AUC of 0.773, 0.757 and 0.806 in the training cohort, internal cohort and external cohort, respectively. The coefficients of the RS model were shown in Fig. S1. The CFRS model achieved an AUC of 0.836, 0.812 and 0.832 in the training cohort, internal cohort and external cohort, respectively. However, compared with the RS model, the CFRS model did not show statistically significant improvement (Delong test P: 0.313 [internal validation cohort], 0.372 [external validation cohort]).

The DL model constructed without SE module achieved an AUC of 0.858, 0.835 and 0.837 in the training cohort, internal cohort and external cohort, respectively. The CFDL model achieved an AUC of 0.907, 0.898 and 0.873 in the training cohort, internal cohort and external cohort, respectively. However, compared with the DL model, the CFDL model did not show statistically significant improvement (Delong test P: 0.134 [internal validation cohort], 0.219 [external validation cohort]).

**Fig. 3:**
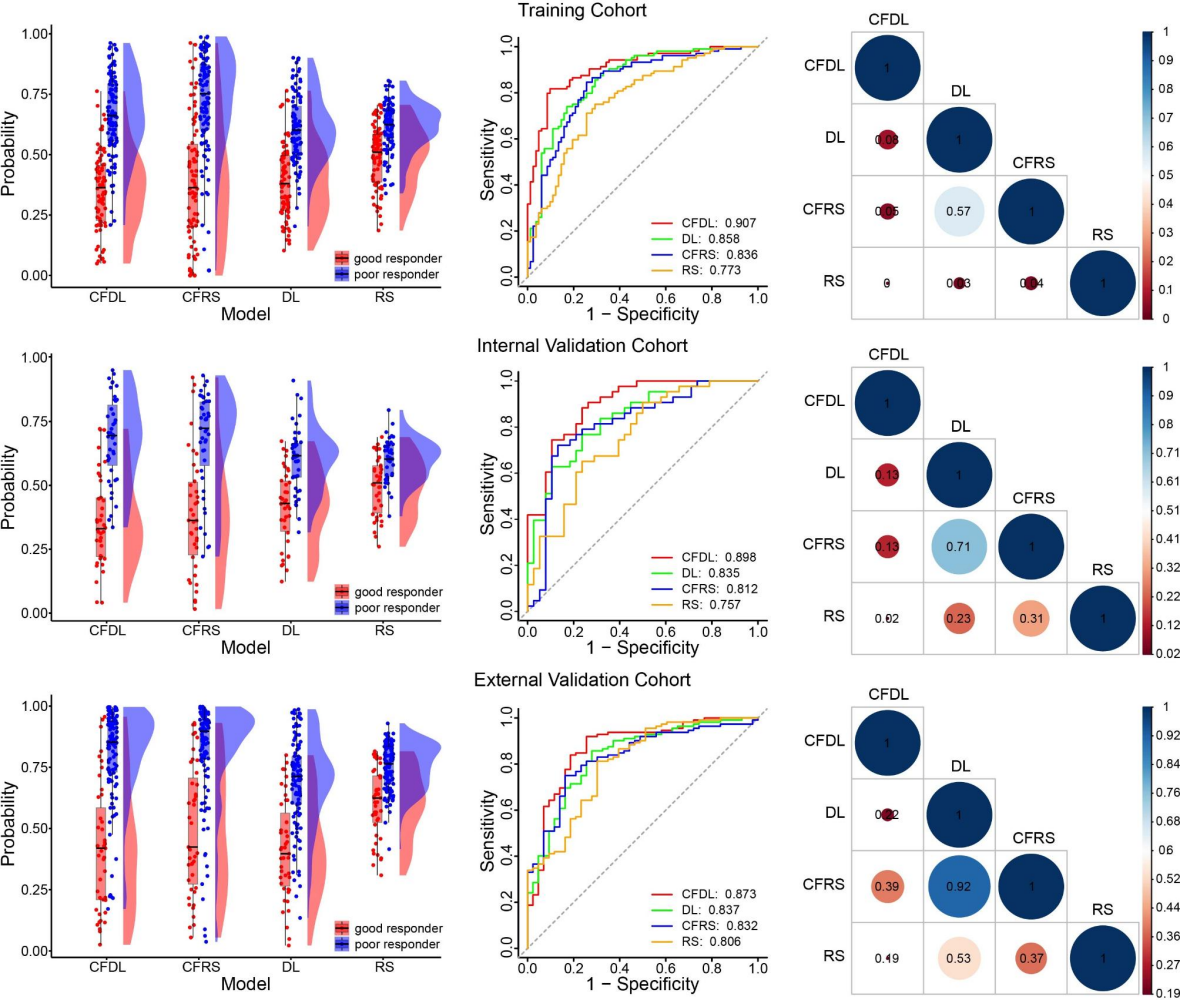
The performance of four models in the training, internal validation and external validation cohorts. The distribution of probability values of patients predicted as poor responders (the left column); the receiver operating characteristic curves (the middle column), p-values for Delong test (the right column)

**Fig. 4:**
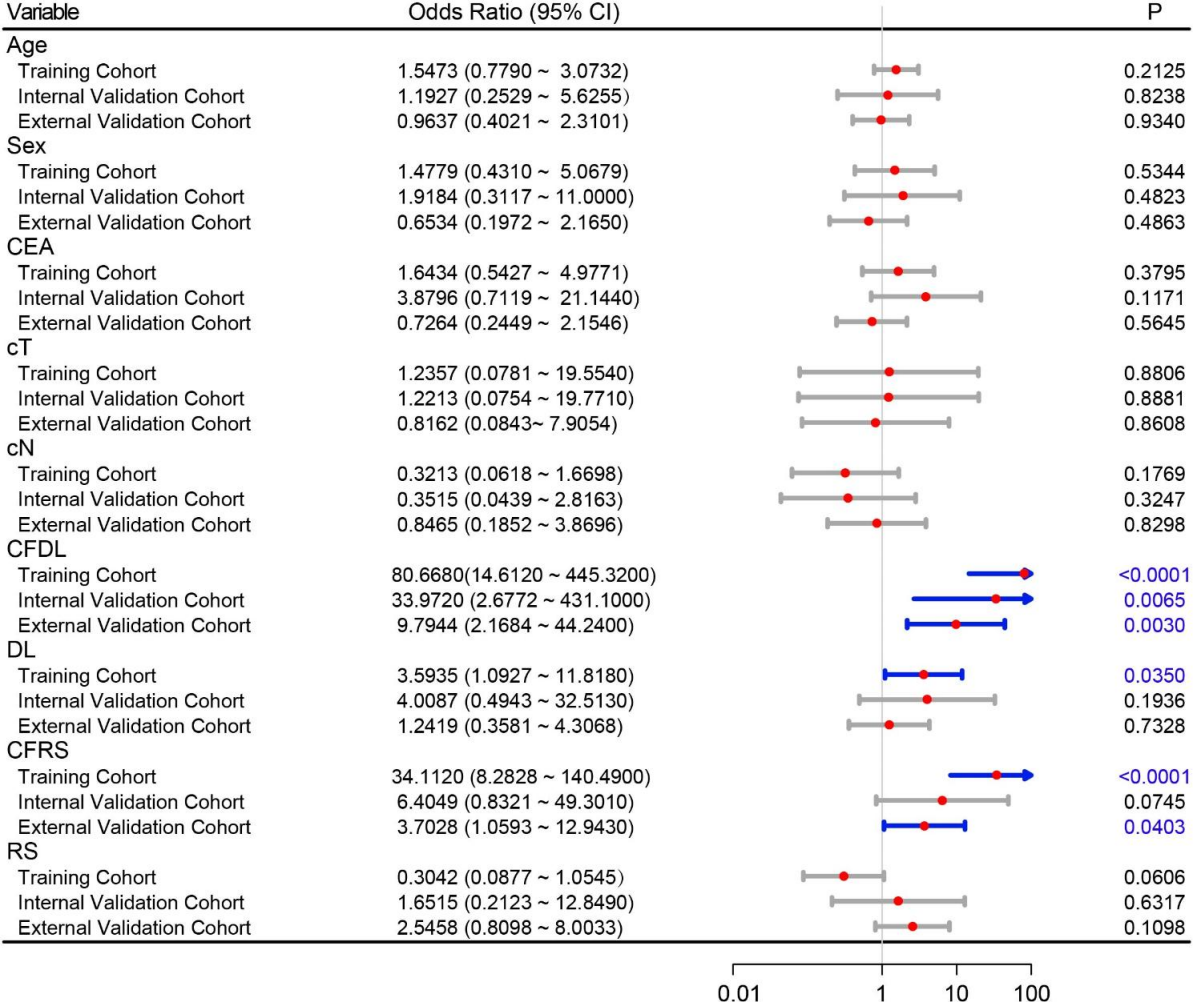
**Odds ratio of clinical variables and developed models in multivariate logistic regression.** CEA, carcinoembryonic antigen; cT, clinical T stage; cN, clinical N stage

Compared with the RS model, the CFDL model showed a significant better performance in both the training and internal validation cohorts, while that was not significant in the external validation cohort. The distribution of probability values of patients predicted as poor responders was shown in Fig. 3**(the left column)**, the ROC curves of the four models were depicted in Fig. 3**(the middle column)**, and the p-values for Delong test were also recorded in Fig. 3**(the right column)**. The values of sensitivity, specificity, positive PPV, NPV and accuracy were listed in Table 2. We also investigated if the combination of CFDL and CFRS can yield better results. The results showed that the combination of the 2 didn’t perform better. In the internal validation cohort, the combined model achieved better accuracy compared with CFDL (83.9% VS. 77.8%). However, in the external validation cohort, combined model achieved very close accuracy compared with CFDL (85.8% VS. 86.5). The detailed results were shown in the Fig. S2. Multivariate logistic regression analysis indicated that the result predicted by CFDL was the only highly significant (p < 0.05) predictive factor in all three cohorts, as shown in Fig. 4.

**Table 2:** Performance index of different models in two validation cohorts

Cohort	Model	AUC	Accuracy	Sensitivity	Specificity	PPV	NPV
**Internal Validation**	**CFDL**	0.898 (0.834–0.964)	77.8% (68.8-87.0%)	76.7% (64.3-89.9%	78.9% (65.8-91.9%	80.5% (68.2-92.9%	75.0% (61.3-89.1%
	**DL**	0.835 (0.745–0.921)	75.3% (65.4-84.8%)	76.7 (63.9-89.1%)	73.7% (58.9-87.9%)	76.7% (63.5-89.6%)	73.7% (59.4-87.3%)
	**CFRS**	0.812 (0.711–0.916)	76.5% (67.3-85.9%)	76.7% (64.2-89.7%)	76.3% (62.7-89.8%)	78.6% (65.7-91.2%)	74.4% (60.8-88.5%)
	**RS**	0.757 (0.648–0.869)	69.1% (59.1-79.3%)	67.4% (53.1-81.7%)	71.1% (57.0-85.4%)	72.5% (58.6-86.3%)	65.9% (51.5-80.4%)
**External Validation**	**CFDL**	0.873 (0.806–0.938)	86.5% (81.1-91.7%)	91.9% (86.9-96.9%)	72.1% (58.8-85.2%)	89.6% (84.1-95.1%)	77.5% (64.3-90.2%)
	**DL**	0.837 (0.764–0.910)	80.0% (73.7-86.5%)	83.0% (76.3-90.2%)	72.1% (58.4-85.8%)	88.6% (82.2-94.9%)	62.0% (48.5-76.2%)
	**CFRS**	0.832 (76.6-89.9%)	79.4% (72.9-85.9%)	89.3% (83.5-94.9%)	53.5% (39.1-69.9%)	83.3% (76.6-90.4%)	65.7% (49.8-81.5%)
	**RS**	0.806 (0.731–0.883)	81.3% (75.1-87.6%)	97.3% (94.4-100%)	39.5% (24.8-54.8%)	80.7% (74.0-87.6%)	85.0% (69.8-100%)

Data are given as value (95% confidence interval)

PPV, positive predictive value; NPV, negative predictive value

### Visualization

**Fig. 5:**
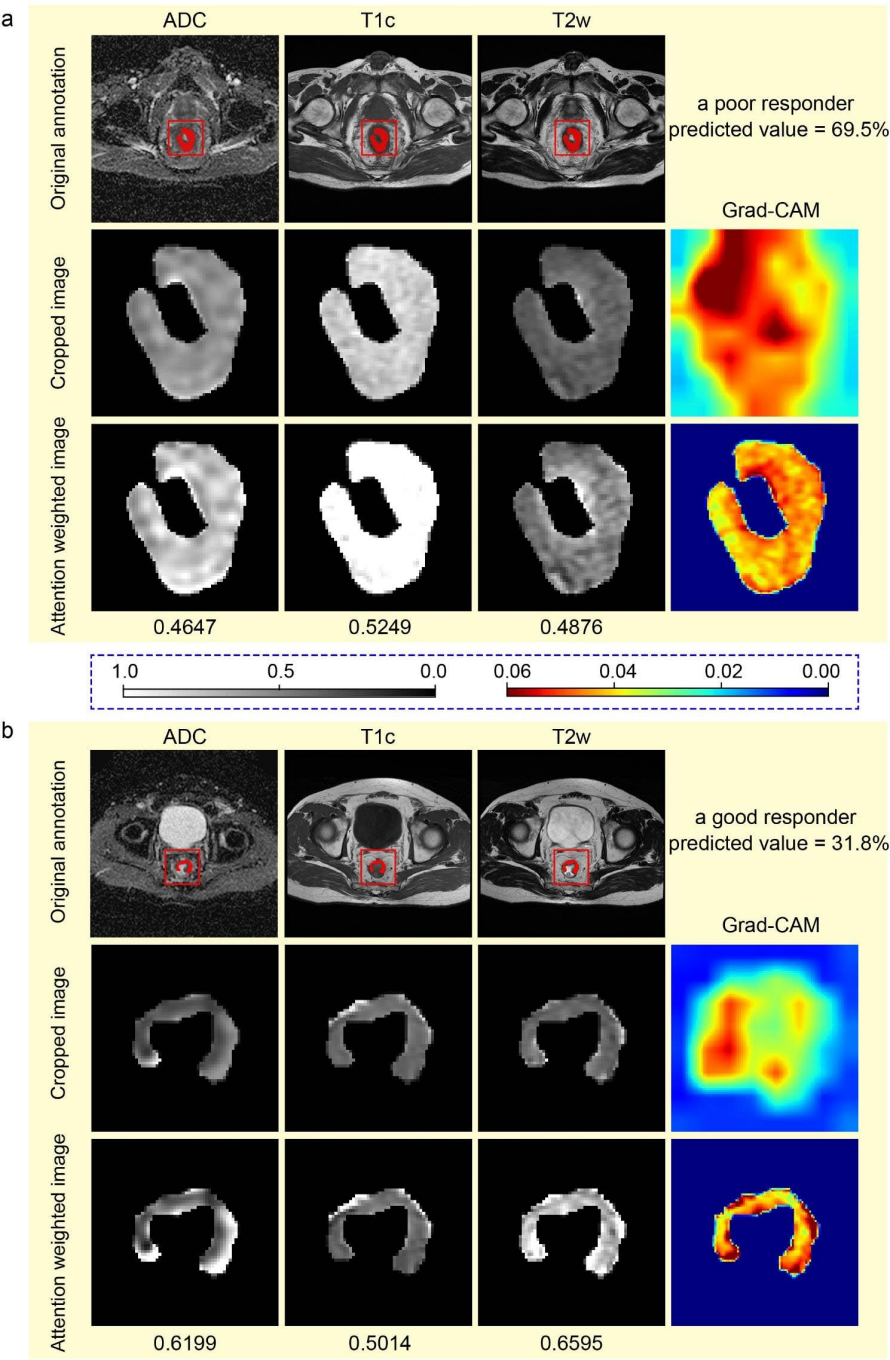
**Grad-CAM heatmap of the last max pooling layer and the first SE-block of the CFDL model in two typical samples.** (**a**), visualization of a poor responder. (**b**), visualization of a good responder. The heatmaps in the second row of (**a**) and (**b**) belong to max pooling layer, and the heatmaps in the third row of (**a**) and (**b**) belong to SE-block.

Two typical sample cases analyzed by the CFDL model were visualized in Fig. 5, of which a true poor responder was predicted to be a poor responder with a probability of 69.5% and a true good responder had a lower predicted probability of being a poor responder. The red area of the Grad-CAM heatmap indicated high probability of being poor response. Therefore, the Grad-CAM heatmaps also indicated that the most tumor area of a true poor responder had poor response and only partial area of a good responder had poor response.

## Discussion

Prediction of response to nCRT in LARC has always been a hotspot of clinical research [11, 24, 25]. In the past decade, radiomics and deep learning have promoted the study of imaging markers for response prediction. This study is an extension of the research field of response prediction in LARC. Two hand-crafted radiomics models and two end-to-end deep learning models were established and validated in an independent external cohort. The results showed both the radiomics and deep learning were promising methods. By comparison, deep learning radiomics performed better than hand-crafted radiomics in the present study.

Recently, Giannini et al. collected pretreatment Positron Emission Tomography and MRI data from 52 LARC patients, and built a radiomics signature combining the two modalities to predict poor responders with an AUC of 0.86. However, this radiomics signature was not validated in another group of patients [26]. Petresc et al. collected pretreatment T2w MRI data from 67 single-center LARC patients, and built a radiomics signature using 44 patients’ MRI and validated this signature using the other 23 patients with an AUC of 0.80 [27]. Shayesteh et al. also conducted a single-center radiomics study with a sample size of 98 and estabished an ensemble learning model to combine four radiomics signature built by four machine learning algorithms. This ensemble learning model achieved an AUC of 0.95 in internal validation dataset [28]. Therefore, this multicenter study was designed to perform both internal and external validation for the developed models. The results showed the four developed models had stable predictive ability in all cohorts.

Many studies have demonstrated that radiomics signature or deep learning radiomics from joint multiparametric MRI performed better than that from the single modality [29–31]. Because different imaging sequences reflect different aspects of tumor biology including tumor intensity, cellularity and vascularization, the combination of multiparametric MRI might improve prediction [32]. Li et al. demonstrated their multi-modal radiomics model that combined Computed Tomography and MRI (T1c, T2w and ADC) features was associated with better performance than any individual sequence [33]. Our previous study also confirmed this conclusion, but with limitation that the therapeutic regimens of this cohort were not very consistent [16]. Based on the above study, the data collection criteria of the present study were strictly determined. Multiparametric MRI was used for modeling, and the parameters for MRI acquisition were consistent within each cohort. The results indicated that either radiomics models or deep learning models had good generalization performance, even though the MRI device version and field strength were both different between the two centers.

Multiparametric MRI and attention mechanism opened up the opportunity to fuse the different sequences to further improve the accuracy over current modeling algorithms. Because of attention mechanism having a great feature selection ability, it has been successfully used in image segmentation [17] and classification [18]. SE network, a lean but efficient self-attention model, that adaptively recalibrated channel-wise feature responses by explicitly modeling interdependencies between channels encouraged the aggregation of multi-channel information in the presence of useless or redundant information from multiple channels. Moreover, SE-based channel-attention module can be easily migrated to other models and does not change the original model structure. In view of the aforementioned advantages, the SE-based channel-attention should be appropriate for deep learning model construction using multiparametric MRI for treatment response prediction, but there are few similar studies published. The present study developed two image-based deep learning models that called DL and CFDL, and confirmed the hypothesis that SE-based channel attention mechanism can improve the predictive ability compared with attention-free model. Inspired by image-based SE module, the present study designed a simple radiomics features based self-attention fusion algorithm called CFRS. The results support that attention mechanism applied to radiomics study can also improve the predictive ability compared with feature concatenation based model.

Most radiomics studies always built a radiomic nomogram with both radiomic signature and clinical variables, expecting a more predictive model than single radiomic signature. The present study analyzed five clinical variables in two independent cohorts, and found that none of these clinical variables was statistically significant predictive in univariate analysis. This result came as no surprise, because many previous clinical studies have analyzed these variables but no robust predictive factors have been identified [34–36]. In multivariate analysis, there still no significant clinical variable was found, whereas, CFDL was a stable predictive factor. This may indicate biomarker derived from using advanced intelligent image analysis method is promising to be a complementary method for treatment response prediction.

This study had some limitations. The first limitation involved the limited sample size and retrospective data collection. Accordingly, the developed model should be validated in larger well-designed prospective studies, which would also enable the collection of more patient and tumor-specific clinical information for developing a more stable and more accurate model. Second, the ROIs were delineated in one single slice, which might not be representative of the entire tumor. Third, more complex and advanced self-attention methods should be adopted, which may further improve the prediction performance. Fourth, the two cohorts differ in terms of outcome and we cannot exclude the presence of a bias. Fifth, the MRI devices were quite different and a more detailed exploration of the role of the technology in the deep learning radiomics versus hand-crafted radiomics performance would be necessary. Sixth, we did not explore the opposite direction, by switching the two hospitals in building training/validation and testing models or by a joint model, obtained shuffling the two set of MRIs and then splitting a training and a testing set.

In summary, this study demonstrated attention mechanism based multi-sequence fusion method was effective for nCRT response prediction in LARC, and improved prediction performance than hand-crafted radiomics method.

### Electronic supplementary material

Below is the link to the electronic supplementary material.

Supplementary Material 1

## Data Availability

The datasets used during the current study are available from the corresponding author on reasonable request.

## Abbreviations

AJCC: American Joint Committee on Cancer
AUC: Area Under the Curve
CEA: Carcinoembryonic Antigen
CFDL: Channel Fusion Deep Learning
CFRS: Channel Fusion Radiomics
CNN: Convolutional Neural Networks
DL: Deep Learning
LARC: Locally Advanced Rectal Cancer
nCRT: Neoadjuvant Chemoradiotherapy
ROC: Receiver Operating Characteristic
ROI: Region of Interest
RS: Radiomics
SAHSYU: Six Affiliated Hospital of Sun Yat-sen University
SE: Squeeze-and-Excitation
TME: Total Mesorectal Excision
TRG: Tumor Rregression Grade
ZCH: Zhejiang Cancer Hospital
