# Supplementary material for: Attention mechanism based multi-sequence MRI fusion improves prediction of response to neoadjuvant chemoradiotherapy in locally advanced rectal cancer
Source: Radiat Oncol. 2023 Oct 27;18:175. doi: 10.1186/s13014-023-02352-y (PMC10612200; doi:10.1186/s13014-023-02352-y)
Supplement: Supplementary file 1 — Supplementary Material 1 [file 13014_2023_2352_MOESM1_ESM.docx]

Table S1. The technical MRI parameters of the scanning sequences.

| Scanner | Sequence | b value (s/mm^2^) | TR  (ms) | TE  (ms) | Flip Angle | Matrix | Pixel Spacing (mm^2^) | Slice Thickness (mm) | Slice Gap (mm) |
| --- | --- | --- | --- | --- | --- | --- | --- | --- | --- |
| GE 1.5T  (Optima MR360) | DWI | 0/800 | 4600 | 85 | 90^。^ | 256 × 256 | 1.10 × 1.10 | 5 | 6 |
|  | T1c | - | 680 | 13 | 90^。^ | 512 × 512 | 0.55 × 0.55 | 5 | 6 |
|  | T2w | - | 4300 | 120 | 90^。^ | 512 × 512 | 0.55 × 0.55 | 5 | 6 |
| Siemens 3.0T  (verio) | DWI | 0/800 | 8700 | 70 | 90^。^ | 130× 170 | 2.2 × 1.8 | 5 | 6 |
|  | T1c | - | 4.6 | 2.0 | 9^。^ | 180× 256 | 1.6 × 1.2 | 5 | 6 |
|  | T2w | - | 5500 | 90 | 130^。^ | 224× 340 | 1.3× 0.9 | 5 | 6 |

*DWI* diffusion-weighted imaging, *T1c* contrast-enhanced T1-weighted, *T2w* T2-weighted.


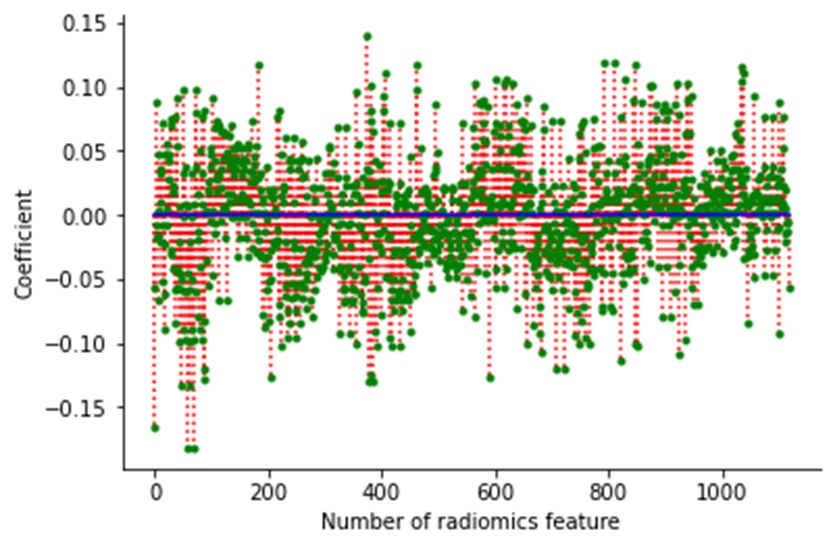


Fig. S1 The coefficients of radiomics model. The blue line indicates zero.


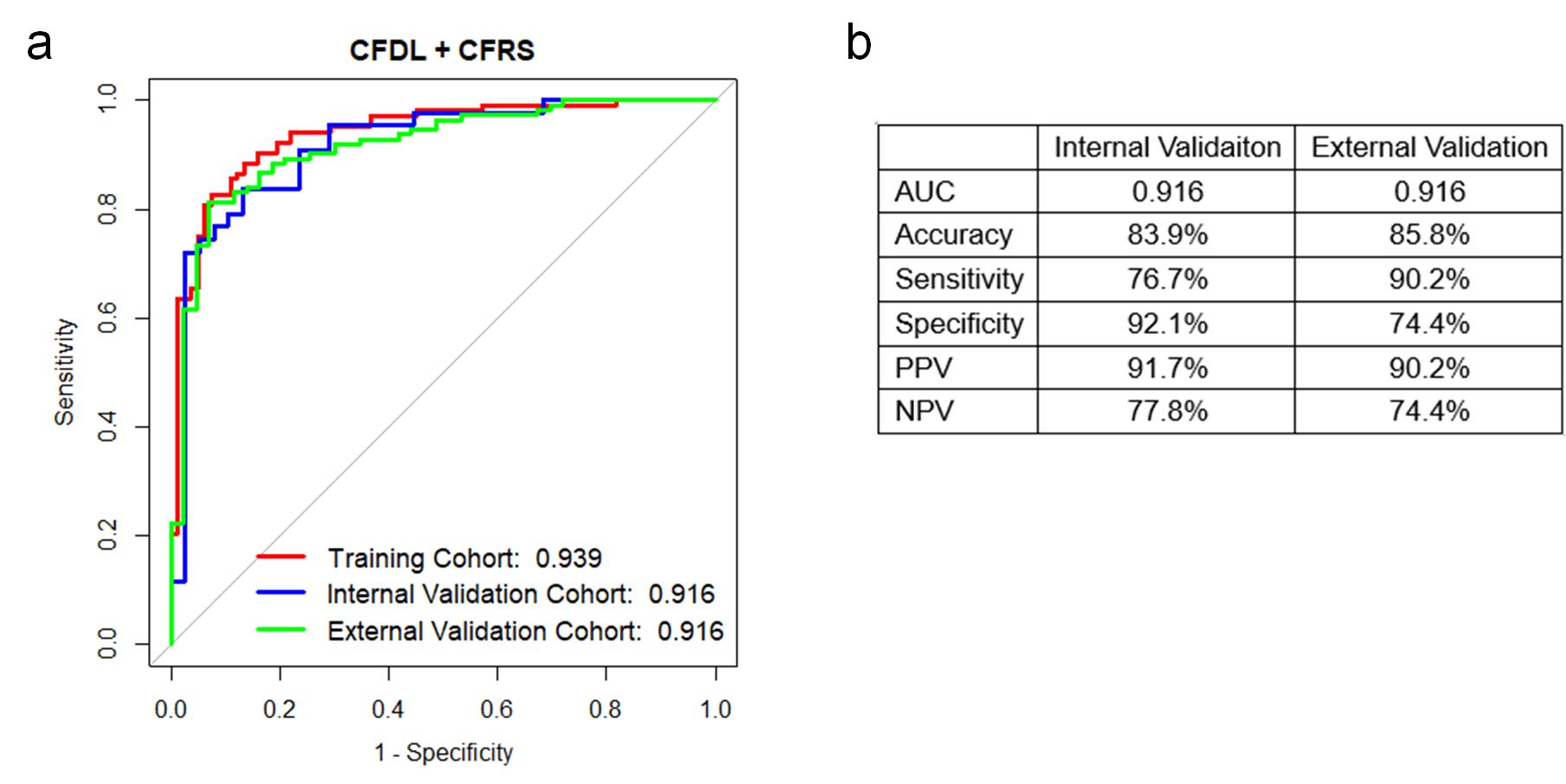


Fig. S2 The performance of the combination of CFDL and CFRS. (a), ROC curves of the combined model in the three cohorts. (b), Performance index of the combined model in two validation cohorts.
